# Evidence-Based, High-Intensity Exercise and Physical Activity for Compressing Morbidity in Older Adults: A Narrative Review

**DOI:** 10.1093/geroni/igz020

**Published:** 2019-07-26

**Authors:** Richard A Winett, Aaron M Ogletree

**Affiliations:** 1 Department of Psychology, Virginia Tech, Blacksburg; 2 Health Research and Evaluation, American Institutes for Research, Washington, District of Columbia

**Keywords:** Compress morbidity, Disablement, Strength, Cardiorespiratory fitness, Resistance training

## Abstract

Recent research in exercise science has important applications for middle-aged and older adults and points to how the programming of individual and multicomponent interventions including theory-based health behavior change strategies may be improved to compress morbidity by delaying or reducing the disabling process. High-intensity interval training and sprint interval training until recently were seen as only applicable to athletes. But recent lab-based research has adapted these interventions for even older adults and demonstrated their safety with beneficial outcomes on cardiometabolic risk factors comparable to or surpassing the usual lower- to moderate-intensity endurance training, and their potential translatability by showing the efficacy of much lower duration and frequency of training, even by systematic stair climbing. Moreover, people report positive affect while engaged in such training. For a century, resistance training was conceived as weightlifting with heavy weights required. Recent research has shown that using a higher degree of effort with lighter to moderate resistance in simple, time efficient protocols result in gains in strength and muscle mass similar to heavy resistance, as well as improvement of cardiometabolic risk factors, strength, body composition, and cognitive, affective, and functional abilities. More effort-based resistance training with moderate resistance may make resistance training more appealing and accessible to older adults. A key potential translational finding is that with correct technique and a high degree of effort, training with inexpensive, portable elastic bands, useable virtually anywhere, can provide appreciable benefits. More emphasis should be placed on long-term, translational interventions, resources, and programs that integrate interval and resistance trainings. This work may improve public health programs for middle-aged and older adults and reflects an emerging evidence base.

**Translational Significance**

A growing research base has shown the critical role of cardiorespiratory fitness and strength for reducing the risk of morbidity and premature death in lab-based settings for older adults. This research provides a viable basis for exercise programs by showing that brief, higher intensity interval, sprint, and resistance trainings can be performed two to three times per week with minimal equipment and are effective for improving cardiometabolic health and strength while also improving cognitive and affective functioning. Such exercises are associated with minimal adverse events and are not perceived as aversive by participants. This developing research base can be the foundation for a range of programs for older adults in community settings and at home.

The primary objective of this article is to perform a focused narrative review from our perspective of current advances in exercise science that can contribute to active aging and the public health goal of compressing morbidity (1,2). The goal of compressing morbidity is not necessarily to extend life. Rather the goal is to extend the quality of healthy, active life and reduce health burden and costs by delaying the onset of chronic diseases and disabilities through prevention approaches or early intervention (1,2).

The key is “effective.” What do we know from exercise science about protocols that have multiple favorable effects and can, at a minimum, augment usual interventions for older adults such as the Diabetes Prevention Program (3) where the prime exercise is usually lower- to moderate-intensity walking?

One reason for this review is that there have been considerable advancements in some areas of exercise science in the last 5 years, many highlighted and reviewed in the revised United States Physical Activity Guidelines, and some receiving less attention (4). However, there have been mixed outcomes in a highly funded, multisite, high-dose intervention for older adults at risk for major mobility disability (5,6), such as the LIFE intervention. At a minimum, these outcomes point toward examining the bases of interventions and other evidence-based alternatives and necessary prevention and treatment research and translation efforts.

This review is not suggesting that any one of the newer approaches represents a panacea and that masses of people will immediately embrace these new approaches. Rather, the findings are presented in the spirit of “promising developments” particularly with the exercise content of interventions. It also should be clear that these evidence-based approaches to exercise will not be readily initiated or maintained without accompanying sophisticated, theory-based approaches to behavior change (7). This review primarily focuses on one side of the translation equation—the actual content and required behaviors of these interventions—and also describes necessary research.

## Theoretical and Conceptual Guidance

This review is guided by two frameworks for understanding the relationship between physical activity and outcomes such as morbidity and mortality: the disablement process (8) and social cognitive theory (SCT) (7,9).

**Figure 1.**
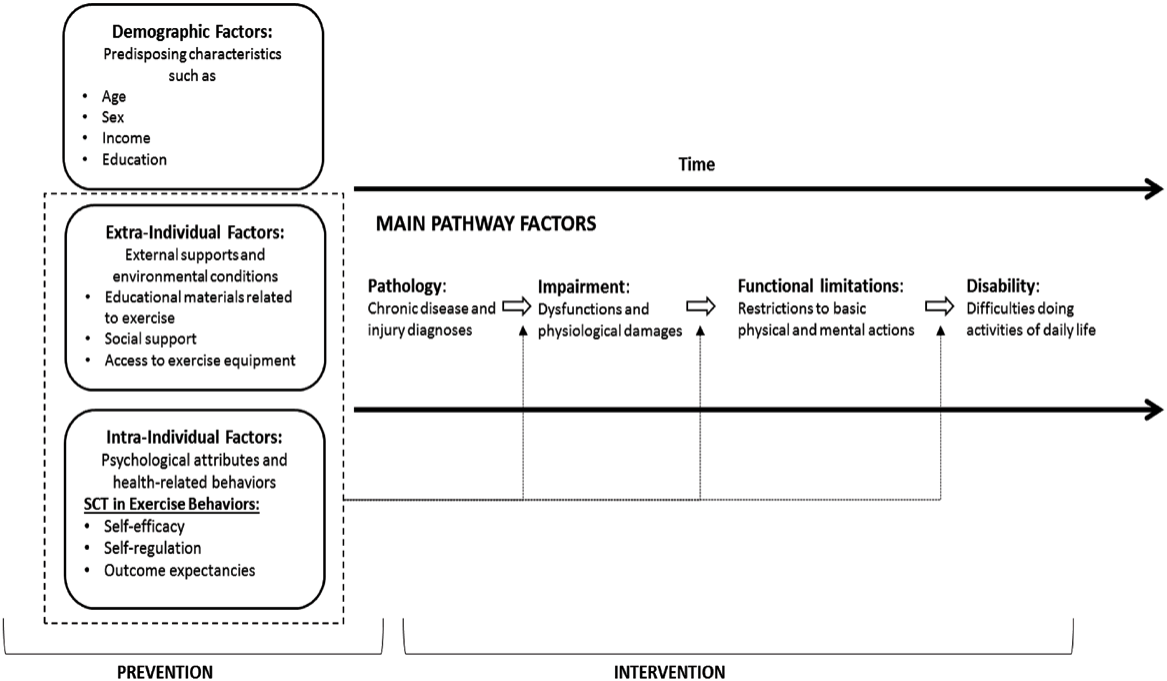
The disablement framework with social cognitive theory for exercise. Adapted from Verbrugge and Jette (8).

The disablement process (8) shown in Figure 1 outlines the progression of illness onset or injury occurrence through impairment (ie, physiological damages), functional limitations (ie, restrictions in basic abilities), and possible disability (ie, difficulties doing daily activities). Within this pathway, factors that predispose older people to morbidity and disease progression include (a) demographic factors such as age, sex, and income; (b) extraindividual factors that facilitate or inhibit exercise such as access to educational materials and equipment; and (c) intraindividual factors, such as health behaviors, psychological attributes, and motivation (10). The latter of these, extraindividual and intraindividual factors, are potentially modifiable and present an opportunity to promote exercise interventions. Thus, the focus of our application of the disablement process is to illustrate the utility of exercise interventions in both preventing the onset of chronic illness and intervening to disrupt the progression of chronic illness on the pathway to disability. To complement this focus, we also use SCT to understand and enact psychological mechanisms that influence the effectiveness of exercise interventions.

As displayed in Figure 1, we have embedded components of SCT within the disablement process framework to demonstrate their complementarity. Consistent with Bandura’s elaboration of SCT (9), Lachman and colleagues noted that self-efficacy (belief in the ability to perform a behavior to produce a specific outcome), self-regulation (eg, planning, focused attention, self-monitoring, and setting goals), and outcome expectancies (beliefs about positive outcomes or avoidance of negative outcomes from performing the behavior) are essential for establishing and maintaining positive health behaviors.

Our adapted version of the disablement process model (Figure 1) focuses on promoting effective exercise programs via extraindividual and intraindividual factors to prevent the onset of chronic illness and mortality and intervene in the disablement pathway to avoid disability. We demonstrate this with the use of dashed lines around modifiable factors that might prevent the onset of disease and also to point to opportunities for prevention along the main pathway. Together, this framework highlights sensitizing concepts that are common in physical exercise intervention research and provide an understanding for processes and underlying psychological mechanisms related to effective physical activity interventions capable of compressing morbidity.

## High-Intensity Interval Training and Sprint Interval Training

The mainstay in most health behavior interventions with a physical activity or exercise component (eg, the Diabetes Prevention Program) has been longer duration (30–45 minutes) lower- to moderate-intensity (50%–70% of maximum heart rate) endurance training such as brisk walking. Performing this type of physical activity and exercise most days of the week also is the most basic physical activity guideline (150 min/week (4); see Chapter 1). This approach does have beneficial effects (4) on many mechanisms associated with health and disease prevention and treatment such as cardiorespiratory fitness and insulin sensitivity. Perhaps a basic assumption is that most people, particularly older adults, will not or cannot engage in more intense exercises. Self-report data from the Centers for Disease Control and Prevention suggest that about half the adult population reaches the goal of about 30 minutes of such physical activity most days of the week, and therefore the country is on a healthful trajectory (11). However, objective data show that only about 5% of the adult population meets this basic goal (12), which is inadequate and points to the need to examine the efficacy and potential translatability of other modes of exercise.

It also is known that it is the degree of *intensity* in exercise that is most associated with improvement in many health-related mechanisms such as cardiorespiratory fitness, with *fitness*, not physical activity per se, the strongest predictor of morbidity and premature mortality (13–15) and even health care expenditures (16). Moreover, recent research indicates that while high-intensity interval training and resistance training improve mitochondrial function, moderate-intensity, steady-state training does not have this favorable effect (17). For older adults, training at less than 60% of maximal heart rate may not be of sufficient intensity to improve cardiorespiratory fitness (18,19).

### Protocols

High-intensity interval training was extensively reviewed in the revised Physical Activity Guidelines (4), though additional points and updates are provided here. Two types of high-intensity exercises include high-intensity interval training and sprint interval training. Preceded by a warm-up, high-intensity interval training involves one or more intense work periods followed by an easier interval and then a cooldown to complete a session. Sprint interval training is similar but with typically much briefer sprints that require maximum effort. These two training modalities have been used for close to a century by athletes. The adaptation to nonathletes for enhancing health is relatively new (20). And, typically, nonathletes perform these protocols on a treadmill or a stationary bike, the latter of which can be easily modified for older adults.

The protocol with the most evidence is Wisloff’s Group 4 × 4 protocol with studies performed with diverse participants. After a warm-up, there are four work periods at 85%–90% maximum heart rate of 4 minutes, each followed by an easier work period of three minutes. While it cannot be claimed that this protocol is brief, it is very effective. More than traditional moderate-intensity training like brisk walking, the 4 × 4 improves fitness, all facets of the metabolic syndrome, body composition, and positively affects mitochondria (20,21). These studies show few adverse events even when the protocol has been used in supervised training with older adults with congestive heart failure (22,23). Some data, albeit not systematic data, suggest people prefer the 4 × 4 to the typical, steady-state, moderate-intensity training (24,25), a finding not highlighted in the revised physical activity guidelines (4).

### Cardiac Rehabilitation

Wewage and colleagues (24) reviewed studies on high-intensity interval training compared with moderate-intensity continuous training with patients with cardiovascular disease who were in cardiac rehabilitation programs. The studies in this review had high-intensity interval training protocols with durations of intervals up to 4 minutes, at or above 85% of maximal heart rate (or a comparable measure), and a rating of perceived exertion score of 15 or more on a 20-point scale. The 4 × 4 protocol was the one most frequently used. Moderate-intensity programs were those with a heart rate range of 60%–75% of heart rate peak, and a rating of 12–15 on the scale. Across the studies reviewed, 547 patients performed interval training and 570 performed moderate-intensity training or received usual care. These patients had an average age of about 61% and 83% were men.

Across all the interval training sessions in these studies, there were 17,083 training sessions and 11,333 training hours. For moderate training, there were 14,268 sessions and 11,213 training hours. Across all the studies and training sessions, there were only seven adverse events, with five of those in interval training. Two of these events were cardiovascular related, one major and one minor, and the five other adverse events were not cardiovascular related (eg, musculoskeletal). There were no differences in event rates between interval training and moderate training with both being very low.

There are several considerations for implementing interval training for use with cardiac rehabilitation. Patients must be screened and receive a stress test so that those with severe cardiovascular disease are not included as was the case for studies in the review. Interval training also is likely to necessitate more staff for better supervision, and patients’ preferences regarding type of training also need to be considered. But given the beneficial outcomes for interval training such as an improved level of cardiorespiratory fitness and lower risk of events and mortality, it is not hard to imagine that in the near future high-intensity interval training could be the main exercise component in cardiac rehabilitation.

### Threshold of Intensity

Although there are numerous other protocols with emerging evidence (26–28), an intriguing line of research with considerable practice implications, and not a focus of the revised physical activity guidelines, is based on much briefer protocols. These studies have investigated the question of whether four work periods are necessary. Is it simply reaching a level or threshold of higher intensity for a few minutes that provides the stimulus for health-related changes?

Some research, though not extensive, showed that one 4-minute (1 × 4) work period provided similar benefits to four work periods (4 × 4) when the contrasting number of work periods in the different protocols were performed at the same degree of intensity (29,30). Another larger study showed that the 1 × 4 protocol was as effective as the 4 × 4 protocol in improving the risk factors comprising the metabolic syndrome for adults whose average age was 56 years (31). For many adults, a 4-minute harder work segment could equate, for example, to walking quickly up an extended hill outside or on an inclined treadmill, or a 4-minute higher intensity effort while biking down the street or on a stationary bike. Such 4-minute protocols, perhaps outside of a gym setting, need to be evaluated with older adults under real-world conditions. Importantly, investigations by a number of groups have shown that older adults are responsive to high-intensity interval training and that even performing a protocol every fifth day can improve risk factors (32).

### Caveats About High-Intensity Interval Training

Despite these positive outcomes and consistent with what was reported in the revised physical activity guidelines (4), a number of caveats should be noted. Many of the studies in this area involve supervised training for only 6–12 weeks. Therefore, the degree of long-term adherence and adverse events outside of this context remains unknown until longer translational studies are conducted (see below).

More recent meta-analyses not included in the revised guidelines (4), are, however, generally consistent with the guidelines. In a meta-analysis, Wen and colleagues (33) included 53 studies that assessed high-intensity training and sprint interval training (reviewed below) when different protocols were compared with a control, nontraining condition or with moderate-intensity endurance training. Study participants varied from healthy adults, to overweight and obese adults, and athletic adults. Most of the studies were short term, and apparently, none were focused on long-term maintenance. Compared with nontraining control study participants, any type of high-intensity training protocol showed improvement in cardiorespiratory fitness. However, it was protocols with longer work intervals, that is, greater than 2 minutes, and a longer total work time in the overall protocol, 15 minutes or more, that produced better outcomes. Likewise, it was this degree of volume that produced marginally better outcomes than traditional, moderate-intensity endurance training. As noted above, research on much briefer protocols (eg, the 1 × 4 minutes), though encouraging, is limited.

It appears from this meta-analysis that following the major evidence, the main high-intensity training recommendation should be the well-known 4 × 4 protocol (4 work-parts of 4 minutes at 85%–90% maximum heart rate, with three easy minutes between work-parts). When a warm-up and cooldown are added to the overall protocol, this entire workout can take 35–40 minutes. It is not clear if doing much less than the 4 × 4 has a greater effect than the usual moderate-intensity (35–40 minutes) endurance training, however. Further research on the volume of training is discussed later.

Another three meta-analyses were revealing and important for the clinical and public health goal of compressing morbidity. These meta-analyses examined the effects of high-intensity interval training often compared with moderate-intensity training on a number of health-related outcomes including fitness. Pattyn and colleagues (23) assessed effects on patients with coronary artery disease and heart failure; Liu and colleagues (34) evaluated effects for people with Type 2 diabetes; and Hannah and colleagues (35) assessed effects for people participating in cardiac rehab programs. Training in the studies included in these analyses was generally supervised. Even with these at-risk patients, there were very few adverse events. Compared with moderate-intensity training, high-intensity interval training showed on average about a one-half MET greater increase in cardiorespiratory fitness than moderate-intensity training. This difference, though small, is associated with meaningful reductions in risk for death from cardiovascular disease and about a 22% risk reduction for all-cause mortality (23). Though these results are encouraging, again it is unclear what effects would persist long-term without supervision, and whether over the long-term moderate endurance training groups would accrue the additional degree of fitness afforded by high-intensity interval training.

### Sprint Interval Training

Sprint interval training research for nonathletes has proceeded in a relatively orderly way over a period of more than 20 years. A first impetus was a seminal study applying the Tabata protocol, which was previously used to train Olympic athletes (36). The key to sprint interval training, as noted, is all-out maximum effort. This first and subsequent protocols featured a series of 20-second sprints or 30-second sprints (“Wingates”). As the term all-out indicates, these are hard, very intense protocols, particularly hard when there is minimum rest between sprints. For example, the original Tabata protocol featured 8–10 all-out, 20-second sprints, with only 10 seconds between sprints (33). Indeed, it is difficult to imagine many middle-aged and older adults performing this original protocol.

However, a remarkable progression has occurred through a series of studies by asking a simple question. What is the most minimum stimulus required to produce clinically meaningful effects on cardiorespiratory fitness, insulin sensitivity, and mitochondria? The answer is about 1 minute of very high-intensity effort within each of three weekly workouts or 3 minutes per week.

The protocol developed by Gibala’s group involves a 3-minute warm-up, three 20-second all-out sprints *separated by 2 minutes of easy work*, and a 2-minute cooldown, for a total time commitment of 30 minutes per week (37). The protocol produced similar effects on cardiometabolic measures at a fraction of the work time than the traditional 40-minute moderate-intensity, steady-state endurance training protocol (37). Other research has indicated that two all-out sprints can provide a sufficient stimulus with 20-second sprints but not with 10-second sprints (38).

Yet another protocol that has been investigated that is midway between high-intensity interval training and sprint interval training is the 5 × 1 minute protocol (39). After a brief warm-up, five 1-minute sprints are performed though not at maximum intensity, with 90 seconds between repeats. Performed three times per week, this time-efficient protocol produced results for cardiorespiratory fitness and glycemic control similar to the much longer typical endurance training.

Perhaps of most importance for potentially translating sprint interval training into public health practice are newer studies not reviewed in the revised guidelines (4). The Gibala group has shown that the stimulus for this sprint interval protocol can be provided through rapid stair climbing (40), and perhaps even with an hour or two between 20-second stairway sprints, an approach called “exercise snacks” (41). While focusing on safety (eg, keeping a hand on the railing), an office, apartment building, or even home stairways can be “sprint interval centers” for many middle-aged and older adults. This is particularly useful for older people who may face extraindividual barriers (see Figure 1), such as not having access to formal workout equipment. Alternatively, and perhaps even safer, sprint interval training can be performed on a recumbent, stationary bike with a person fully seated with back support. These bikes sit lower to the ground than traditional stationary bikes, which makes mounting and unmounting the bike less risky and less reliant on balance and coordination. The key is the high degree of intensity, but now made seemingly more feasible by only two to three repetitions with 2 minutes (or perhaps, longer) between repetitions. Clearly, these are translational studies that need to be performed with older adults, especially, to assess safety and longer-term maintenance of the protocol in the natural environment.

With regard to translational research, Wisloff’s group in Norway recently demonstrated in a research study (42) with about 300 healthier older adults that with some initial instruction and a few sessions of supervised training, participants had good adherence to unsupervised high-intensity interval training over the course of 1 year, meeting the goal by averaging two such sessions per week. Adherence was assessed by paper or digital logs with the goal to (a) adhere to the protocol, (b) reach the higher level on a rating of perceived exertion scale, and (c) in a location of performance (eg, outdoors) based on personal preferences.

As Hunter and colleagues noted: “. . . it is possible for a highly fit 75 year old to have similar physiologic function to a 35 year old . . . However, the key is intensity. Without at least a small amount of high-intensity training, the progressive loss of muscle function and weight gain will accelerate the deleterious effects of aging” (43). Indeed, adopting exercise practices that incorporate high-intensity activities *may* delay the onset of morbidity and mortality better than low- to moderate-intensity protocols.

### Objections

The major objections to high-intensity interval training and sprint interval training are that these protocols presumably produce high numbers of adverse events and that people will find their performance aversive and, therefore, discontinue high-intensity training. However, the 4 × 4 protocol, as noted, has been used in a supervised training context with older adults who have congestive heart failure or otherwise are in cardiac rehabilitation or, as noted above, have other major chronic diseases, with very few adverse events occurring (22). Importantly, the revised American College of Sports Medicine risk stratification algorithm makes participation in higher intensity training more likely and indicates some older adults without major risk factors can engage in this activity with modest medical clearance, and some without medical clearance altogether (43). For these adults without major risk factors, high-intensity interval training may be effective in delaying the onset of a chronic diseases while also preventing the progression of disability as described in the disablement process model. It is also important to note that recent research has affirmed the highly protective effects of cardiorespiratory fitness on mortality (44).

Moreover, a recent scoping review involving 42 studies with both previously active or inactive, healthy or unhealthy participants focused on the experience of performing these high-intensity protocols (24). Measures of affect and enjoyment in these studies indicated similar or more positive ratings for participants involved in high-intensity training compared with participants performing conventional, moderate-intensity steady-state training—a finding not in the revised physical activity guidelines (4). This area of research needs to expand to assessing a full range of social cognitive variables (7,9) over a long course of more typical, unsupervised training (42–44), especially with middle-aged and older adults, within a program of translational research (45,46).

### Cardiorespiratory Fitness

A review by Harber and colleagues of research since 2009 presented an even more compelling picture of the importance of improving cardiorespiratory fitness through high-intensity activities (47). In the many studies that were reviewed, fitness was measured in various ways and sometimes estimated using established equations. Across different age and demographic groups, healthy and unhealthy adults, cardiorespiratory fitness had a profound impact on heart disease, cancer risk, and risk for premature death. Chronic illnesses such as heart disease and cancer are also related to higher levels of unmet need among older adults, which often leads to a “watershed” of adverse events such as falls (48) and hospitalization (49). Thus, cardiorespiratory fitness is effective in delaying and perhaps avoiding the onset and progression of such illnesses and injury, which lead to functional limitations and premature death. What also is striking is that the largest risk reduction is afforded by minimally increasing fitness (ie, 1.0–1.5 METs) so as not to be in a low fitness group (lower 20%) for one’s age and gender (50). Cardiorespiratory fitness seems to encompass the coordination of different physiological and neuromuscular systems to perform at a high degree of effort, thus providing a basis for its prediction power.

There now is a strong movement to assess fitness as a vital sign in primary care and more specialized health care using evidence-based prediction equations (ie, estimated cardiorespiratory fitness (51)). As assessing fitness in health care evolves, it is important that providers appreciate the profound beneficial effects of exercise, are at least conversant with developments in this area for risk stratification (43), and can recommend to patients an evidence-based approach within their capabilities and preferences such as high-intensity interval training walking (52) or systematic stair climbing (40), and then carefully track the behavior and provide instruction and feedback (7,9).

### Moderate Intensity

However, even when performing high-intensity training, there still appears a need to perform some moderate-intensity training. A recent study focused on a different and quite important mechanism: arterial stiffness (53). With aging, there often are changes in the vascular system. Arterial stiffness is a thickening and stiffening of large elastic arteries. Arterial stiffness is associated with cardiovascular events and premature death. It was reported that moderate-intensity training improved arterial stiffness but high-intensity interval training did not show improvement (53). The investigators noted that it is not entirely clear what mechanisms would explain these different outcomes for moderate-intensity training and high-intensity interval training. However, the study’s findings suggest that the mechanisms involved in improving fitness, in this study from high-intensity interval training, may not be the same mechanisms to improve arterial stiffness. The study points to middle-aged and older adults continuing physical activity outside of more intense training such as also brisk walking 20–30 minutes several times per week or any comparable activity. Arterial stiffness (53–55) will be addressed again in later sections.

## Resistance Training

Engagement in resistance training was until recently considered a mode of exercise quite secondary to aerobic exercise (56). However, it is now known that in addition to increasing strength, muscle mass, and functioning, engaging in resistance training is also associated with notable cardiometabolic benefits and can decrease risks for cardiovascular diseases, some cancers, and premature death (56–61). These profound and numerous benefits of resistance training for older adults, pointing to resistance training as a prime intervention (56), were minimally noted in the revised physical activity guidelines (4). Thus, this line of work points to important avenues for health improvement and intervention efforts. Recent research also indicates that specific thresholds of strength based on age and gender can predict morbidity (57). The retention of muscle mass and strength and the ability to coordinate strength are critical for remaining functional and avoiding the adverse effects of sarcopenia (62–64), which is consistent with the disablement process framework. Rather than a secondary mode of exercise, a case can be made that for middle age and older adults, resistance training should be the primary mode of exercise, and a major focus for public health (56,59) should be to increase the low prevalence of resistance training across age groups and especially older adults (65,66). Based on the recommendation of training major muscle groups twice per week, the percentage of older adults performing resistance training is only about 5% (67).

As noted previously, the more than century-old paradigm for resistance training, still prominent in some quarters, has been weight lifting. The most basic assumption is that the stimulus primarily involves the amount of weight lifted (ie, the external load) and that generally “heavier is better.” Heavier resistance has been believed to provide the best stimulus for increasing strength, muscle hypertrophy (ie, muscle stimulation and growth), and other favorable cardiometabolic benefits.

### Effort-Based Training

A series of studies by Phillips’ group, not noted in the revised guidelines (4), has shown that this long-standing paradigm is not correct (68). Instead, the stimulus that produces many benefits is defined by a *degree of effort*. In practice this means that light to moderate resistance can provide an excellent stimulus when a set of repetitions is taken to a high degree of effort and where the last repetition that can be performed is in good form (ie, contractile failure or “training to failure” (62)). This is not to suggest that such resistance training is “easy,” but rather that the use of lighter to moderate resistance may make resistance training safer and more accessible and appealing to a wider spectrum of the population. Thus, the adoption of such exercise among older people is feasible and may be used to intervene or slow the progression of disabling events.

The caveat is that simply “going through the motions” may only provide minimal benefits. Training with lighter to moderate resistance with a high degree of effort produces muscle hypertrophy outcomes similar to using heavy resistance and meaningful strength increases (69). This is important because strength is inversely associated with morbidity and mortality (67,68). Moreover, in the sarcopenia pattern (loss of muscle mass and strength), it appears that loss of strength is most critical for difficulties in functioning (64) and subsequent disability.

Interestingly, the issue of arterial stiffness has most recently been assessed with resistance training. Findings show that if aerobic training is performed after resistance training, arterial stiffness is improved (54), and, moreover, that resistance training alone, perhaps the effort-based approach with moderate resistance, *may* improve arterial stiffness (55). Clearly, this is another area where more research is needed especially with middle age and older adults to evaluate the long-term protective effects of combined aerobic and resistance training under natural conditions.

### Adaptation and Feasibility

Especially significant for older adults is that effective resistance training requires adaptation to a particular stimulus and recovery (eg, quality sleep (70)) from a prior workout in order for the next workout to produce subsequent positive adaptations (71). For example, after training with and adapting to a particular protocol and *not* experiencing delayed onset muscle soreness, then muscle protein synthesis is associated with muscular hypertrophy (65). Constantly changing a protocol or training when not recovered from a prior workout that results in delayed onset muscle soreness can undermine resistance training. The body interprets a new stimulus or soreness and its impact as an injury, and physiological resources are used for repair of the injury (71).

Likewise, another key part of the adaptation and recovery cycle for resistance training is that older adults appear to need more protein than the current recommended daily allowance of 0.80 grams of protein per kilogram of bodyweight especially when resistance training. Studies indicate that middle age and older adults can improve the outcomes of resistance training by consuming 1.2–1.6 g of protein per day per kilogram of bodyweight (72–76). So, for example, older women weighing 168 pounds (76 kg) should eat at least 91 g of protein per day and older men weighing 195 pounds (88 kg) should eat at least 106 g of protein per day over the course of meals and snacks to maximize the benefits associated with resistance training. The combination of resistance training and high-protein, high-quality nutrition within DASH or Mediterranean patterns emphasizing many servings of fruits and vegetables, lean sources of protein, and minimal processed foods for middle-aged and older adults is another important area for translational research (77). However, no research has evaluated at what threshold of training (volume, frequency, and intensity) does additional protein make meaningful differences in strength and muscle hypertrophy outcomes.

Also important for feasibility, it has been found that training with inexpensive and portable elastic resistance bands provides similar benefits to resistance training with free weights or machines (78). This is particularly notable when considering extraindividual factors (eg, external supports/constraints and environmental conditions) within the disablement process model, such as access to exercise equipment which many older people may not have. Proper form and a high degree of effort are still required but effective use of bands can take place in very limited space and at any time (ie, no need for a health club or gym), though it remains important to apply theory-based, behavioral programs for initiating and maintaining the use of resistance bands (7). Resources such as YouTube provide free instructional videos for safely using resistance bands without the supervision that is typical of many of the lab-based exercise studies noted here; such resources may increase opportunities to translate these interventions from lab-based settings to people’s homes.

Whatever the training modality (free weights, machines, bands), resistance training protocols can be simple, time efficient, and safe. For example, in one study with previously inactive older adults (50–69 years) with prediabetes, a protocol with only 12 machine-based exercises for only one set per exercise, taking about 35 minutes twice per week, led to about a 33% reduction in the prevalence of prediabetes (about the same as the Diabetes Prevention Program) and improvements in strength, body composition, and blood pressure (79). The study also showed how an SCT-based approach (7) could guide people from an initial supervised training setting to training on their own for about 1 year in community facilities to maintain resistance training (37). Over the study’s course of about 12,500 workouts, about 8,000 of those workouts unsupervised during maintenance phases, there were only four, nonfatal adverse events (eg, a high degree of muscle soreness). These data suggest that with some initial supervision, resistance training can be an effective and safe exercise modality within different facilities without continued supervision. Furthermore, within the context of the disablement process model, the study showed that by modifying intraindividual factors such as exercise behaviors through the SCT intervention, the researchers were able to reduce the prevalence of prediabetes thereby delaying the onset of a chronic disease and subsequent impairments and limitations.

Simple, brief protocols may not produce the absolute best outcomes (80). Rather, more volume and training three times per week produce better outcomes (81,82). However, a point of diminishing returns (ie, added benefits) is quickly reached. A public health, translational perspective suggests focusing on protocols that produce some meaningful outcomes and that are feasible (79) such as low-volume resistance training and brief sprint interval stair climbing.

## Future Directions for Research

### Revised LIFE Intervention

Consider how these overall findings for resistance training coupled with other findings can change the way interventions have been implemented, for example, to prevent disability in older adults. Other findings point to increasing strength as a primary vehicle for preventing or treating problems such as mobility limitations and disability in older adults (75). Depending on the functional skill and degree of disability, as noted in the revised physical activity guidelines (9), other task-specific training may be included within an intervention to improve functional abilities (83,84).

The LIFE intervention for preventing major mobility disability provides one example of how the focus on whole-body strength can change interventions. Reduced mobility is an important risk factor for morbidity, hospitalization, and premature death. The LIFE study (5,6) was a multisite, randomized clinical trial where 1,635 older adults (ages 70–89) were randomized to either a physical activity intervention or an education-only condition. To be in the trial, people could have some reduced mobility but had to be able to walk 400 m in less than 15 minutes, though they could use a cane and stop (not sit) for brief rests. The intervention lasted 1 year. Two sessions per week were held at centers where people walked and stretched plus performed resistance training, but this was only leg extension and leg flexion exercises with ankle weights. People were also supposed to perform several sessions per week of the total program at home. The education-only group did not have any prescribed physical activity and mostly was provided in sessions with health information and did some upper-body stretching.

The LIFE trial has been noted as a success because the incidence of major mobility disability over the 2.6 years at post-test was lower in the intervention group than in the education-only group (5). However, in actuality, the results were not that striking especially given a “high-dose” supervised training intervention that cost about $4,900 per person. The rates of incident major mobility disability were 30.1% (intervention group) versus 35.5% (education group; number needed to be treated = 18.5), and the rates of persistent major mobility disability were 14.7% versus 19.8% (number needed to be treated = 19.6). This means it cost about $93,000 to prevent an incidental or persistent mobility disability.

Given the intensity (dose of intervention) and costs of LIFE, it should be clear that this potentially beneficial program may not be as efficacious as other programs that include higher intensity training. However, as noted above, this type of physical activity was believed to be easy to maintain and the results should persist, and probably the difference between groups should be magnified as people became even older. However, a 1-year follow-up after the program ended (6) indicated this was not the case. At that point, there were *no differences* between groups either in the amount of physical activity per week or in functional abilities.

There are several ways a LIFE protocol can be revised and possibly lead to superior outcomes. Note that the original intervention had no upper-body training given the tradition of defining disability by the ability to walk 400 m, and unlike functional skills training, was not centered on improving overall strength. Consider, for example, that many older adults can be mobile in a community *if* they have the strength in their upper and lower bodies to get in and out of cars.

A contemporary, multicomponent protocol could first focus on whole-body training with elastic resistance bands that, after initial center-based sessions, can be used at home to improve overall upper- and lower-body strength (78). Also, starting at a center, but then moved to other indoor and outdoor locations, variations of walk-based, high-intensity interval training effectively used with older adults in Japan (48) and other countries can be performed, or walking can be performed outdoors on hilly terrain. It is not clear if separate balance training and stretching would add any additional benefits (4). More contemporary monitoring using technology, with goals, feedback, and guidance within an extended contact intervention guided by SCT (7,9) may also improve adherence once a more intensive part of an intervention ends (45,79).

The original LIFE intervention also did not consider studies on resistance training and functional skills (85,86). What has been found is that resistance training alone can improve functional skills as traditionally assessed in the absence of any functional skills training, particularly in the absence of more severe disability as was the case for LIFE participants. More severe disability may require resistance training to improve overall strength and then very task-specific training (83,84), such as sit-to-stand movements in nursing home residents. Clearly, there is a need to assess the efficacy of a revised LIFE protocol based on the current evidence base.

### Cognitive and Affective Functioning and Other Areas of Application

An additional area of study for resistance training, not described in the revised physical activity guidelines (4), is its effects on cognitive functioning and the structure of the brain in older adults. For example, white matter lesions in the brain develop in older adults as one indicant of a vascular disease and are associated with cognitive decline and falls, with both associated with additional morbidity and increased risk of death (86). It is now recognized that resistance training has many cardiometabolic benefits and may also be effective for preventing and treating vascular disease (56) and, perhaps, slowing the progression of white matter lesions. Resistance training also may improve cognitive functioning and mobility (87,88).

Studies have generally involved whole-body resistance training with supervised training lasting for up to 1 year. Generally, resistance training is performed once or twice per week, and a comparison condition involves toning and balance exercises, and relaxation training. Standard testing is used to assess cognitive function, magnetic resonance imaging is used to assess white matter lesions, and other standard tests are used to assess strength and gait speed. Typically, in these studies (86–89), twice per week resistance training is necessary to show improvement in cognitive and executive function and memory, a slower progression of white matter lesions, and improvement in gait speed. Once per week training is less effective, and the more typical toning and balance interventions show *no* effects on these measures. Aerobic training also improves cognitive function in older adults though differently from resistance training (90).

However, the previously described LIFE intervention showed minimal positive effects on cognitive abilities (91). By way of contrast, as described above, the same whole-body resistance training protocols that improve cognitive and mobility functioning also provide a myriad of other health benefits for older adults. One of these important areas is affective functioning. Recent meta-analyses have shown that resistance training reduces anxiety and depression (92,93) with the outcomes for resistance training surpassing the effects of some common medications.

Another area of importance to middle age and older adults are recent findings, albeit preliminary and in need of further research, that resistance training can decrease systemic inflammation that underlies different chronic diseases (94).

The area of effective weight management also is changing in ways critical for older adults. It has been shown that without either high-intensity interval training or resistance training, during weight loss, resting metabolic rate and energy expenditure in incidental, nonexercise activities decrease (39). These changes set the stage for regaining weight in the form of body fat rather than muscle mass. It also has been found that with traditional weight loss that does not include resistance training, more of the weight lost can be the loss of muscle mass (95), a finding that is critical for older adults who generally are losing muscle mass due to typical age-related physiological changes. Including resistance training as a major component of weight loss typically does not eliminate the loss of muscle mass, but does slow the rate at which that muscle mass is lost (95). Together, these newer areas of research further point toward resistance training becoming more of a centerpiece of a public health focus on active, healthy aging (56).

## Physical Activity and Sedentary Behaviors

A question that remains unanswered is whether chronic sedentary behaviors in people who are fit or physically active are still detrimental to health. One prospective study (96) and an extensive review (97) indicated this is not the case, suggesting more focus on exercise and physical activity and less, for example, on extended sitting typical of much contemporary work. There is, however, uncertainty in this area. Jean Mayer postulated about 60 years ago that there is a threshold of physical activity needed for appetite regulation and the prevention of weight gain. A recent study found that this threshold is about 7,000 steps per day, considerably above the average for most U.S. adults (98).

A new and important area in physical activity is the research on “step reduction,” that is, less than 1,000 steps per day, a much more severe degree of sedentary behavior (99). These studies simulate what happens when a person is sick or injured and physical activity is extremely limited to the most minimum steps per day. For example, in a study with prediabetic, overweight older adults, during a 2-week step reduction phase, compared with baseline, insulin resistance increased, insulin sensitivity decreased, and muscle protein synthesis decreased (96). These are deleterious and expected effects from 2 weeks of inactivity. In studies with younger adults, although similar deleterious effects of inactivity were found, resuming activity returned these measures to baseline levels (99). However, with the older prediabetic adults, 2 weeks of returning physical activity to baseline levels did *not* restore these important health-related measures to their baseline levels (99).

These data provide a serious warning to lay people and medical professionals about prescribing any treatment, unless absolutely necessary, that involves severe reductions in physical activity. Consider, for example, staying in bed for a week with the flu and then not becoming sufficiently active for the next 2 weeks for adequate recovery. The flu can clearly be dangerous, but, perhaps, similar to recommendations for sedentary office workers, a recommendation can be to get out of bed during the day each hour and walk around at home for 5–10 minutes.

With an injury, it may be possible to still exercise but in a different way. For example, for a person with a leg or back injury, it may be possible to perform workouts on an upper-body ergometer or simply sit on an Air Dyne (ie, a combination stationary fan bike that can be controlled with foot pedals, mobile push–pull handles, or both) and only use the handles for the push–pull upper-body movement.

Interestingly, a prior study conducted at the same university with older adults also had a step reduction phase. In an elegant design (100), one leg of each participant was randomly assigned to a resistance training condition. The training involved coming to the lab three times per week and performing single-leg leg presses and leg extensions for three sets each to fatigue using lighter resistance but with a high degree of effort at the end of a set. The results showed the protective effects of this one intervention for muscle protein synthesis and an increase in the trained leg’s strength and muscle mass even while steps were greatly reduced. In the future, it is possible that injured or ill older adults as part of their recovery plan will be prescribed safe, though high-effort, low-load resistance training. Instead of atrophy and a metabolic disruption during recovery, the result in key research studies may include actual improvements in strength and body composition without metabolic disruption.

## Conclusions

This narrative review has primarily focused on the exercise components of interventions and especially emphasizing the effectiveness of higher intensity training for increasing healthy quality of life, improving functioning, and compressing morbidity through delaying or mitigating disability. It has been noted, however, that such interventions are likely not to be well initiated, much less maintained, without the full incorporation of SCT constructs, principles, and strategies (7,75). An SCT framework for tailoring exercise for middle age and older adults has been previously detailed (7). However, based on the current review focused on higher intensity training, additional points are addressed.

As suggested by the range and options for high-intensity interval training, sprint interval training, and resistance training, there is not one protocol or place option. For example, variations of resistance training and some variation of sprint interval training can be performed within an apartment with the most minimal equipment (bands, walking rapidly in place for sprints), a community facility, or in a state-of-the-art health club with the newest, most expensive equipment, and in any setting, exercising alone or with other people. However, through initial face-to-face supervision, interactive technology, or at least instructional videos, it is critical that people learn how to exercise correctly, effectively, and safely. It is just as critical as Hunter and colleagues noted to have careful explanations of “Why intensity is not a bad word” (50).

Within the context of this review, although the studies themselves can appear methodologically complicated to the public, the exercise protocols themselves are *simple* and, for example, may include 12 exercises for one set each with resistance training and stair climbing for sprint interval training. However, to extend a previous review of SCT and exercise (7), the current review emphasizes exercise as embedded within self-regulation: planning and scheduling of each workout with some latitude (hour, days), given life circumstances; keeping track of workouts in some convenient format that provides feedback and using very gradual progressions and setting realistic goals; focusing attention on how an exercise is affecting the body and for resistance training, specific muscle groups, while keeping a good degree of intensity (eg, 16/20 on a rating of perceived exertion scale); and modifying a protocol so that it always is feasible and can result in positive affect given a sense of accomplishment. Thus, an effective approach to exercise interactively incorporates mechanisms to enhance self-efficacy, self-regulation, and outcome expectancies (7,45), ideally within an overall healthy lifestyle.

From a cognitive-behavioral theory and exercise science perspective, ideal exercises can be conceptualized and enacted. It is important to emphasize, however, that there is a paucity of long-term studies to assess adherence, adverse events, and health outcomes related to the programs described in this review. Basic questions remain unaddressed. For example, a major expense in any program is the supervision of training. It is unclear how many supervised training sessions and “faded contacts” are needed to maintain different types of exercise. For example, sprint interval “exercise snacks” on stairs seem feasible and maintainable over months and years, but there are no data from translational research studies to indicate what initial instructional sessions are needed, if periodic checkups are needed, or how they should be most effectively done.

This review points to investing more resources into longer-term translational research as a basis for public health to promote evidence-based exercise and physical activity, especially for middle-aged and older adults, to compress morbidity by delaying or intervening with disability (1,2,8). At present, and unlikely in the future, there is not a medication or even a group of medications (101) that can provide the same benefits as sustained and safe exercise practices. In some quarters, these efforts will be considered as not needed, maybe too expensive, or soon it may be believed that various health problems will be solved by precision medicine (102,103). However, there are compelling reasons to invest more in public health for both prevention and potentially lower-cost, evidence-based, lifestyle treatment (101–105). Consider this one point: the U.S. annually spends mostly on treatment (~97%), and not public health (~3%), a *staggering* $3.2 trillion on health care (106). If our health care spending was the sole and total economy, it would be the fifth largest economy in the world!

## Funding

None reported

## Conflict of Interest

None reported.
